# A prospective study exploring the predictors of response to benralizumab in patients with refractory bronchial asthma

**DOI:** 10.20407/fmj.2020-024

**Published:** 2021-03-20

**Authors:** Masahiro Hirose, Kazunobu Kuwabara, Rieko Kondo, Takahiko Horiguchi

**Affiliations:** Department of Respiratory Medicine II, Fujita Health University, School of Medicine, Nagoya, Aichi, Japan

**Keywords:** Refractory bronchial asthma, Antibody preparation, Anti-IL-5 receptor α monoclonal antibody, Benralizumab

## Abstract

**Objective:**

This study aimed to evaluate the predictors of response to benralizumab therapy in patients with refractory bronchial asthma.

**Methods:**

After 16 weeks of benralizumab therapy, 32 patients with refractory bronchial asthma were assigned to two groups based on the response to treatment as indicated by changes in the asthma control test score (responders and non-responders) and evaluated for clinical characteristics.

**Results:**

Overall, 25 responders and 7 non-responders were identified at week 16. Logistic regression analysis identified a peripheral eosinophil count of >300/μL during benralizumab treatment and a maximal peripheral eosinophil count of >300/μL in the past year as predictors of response.

**Conclusions:**

The predictors of response to benralizumab included a peripheral eosinophil count of >300/μL during treatment and a maximal peripheral eosinophil count of >300/μL in the past year. These findings could improve patient selection and reduce medical costs in the future.

## Introduction

In recent years, the incidence of allergic diseases has increased globally, and bronchial asthma is a representative allergic disease. Drugs for the treatment of bronchial asthma are mainly inhaled and orally administered. Despite adequate treatment with these drugs, some patients with severe refractory asthma experience impaired quality of life and death in some cases. Recently, various new antibody formulations have been developed and launched for the treatment of asthma. These antibody preparations have displayed excellent therapeutic benefits when combined with traditional anti-asthma medications.

However, antibody preparations present some challenges. Although these treatments are expensive, no predictors of response have been established, making it difficult to examine the benefits and drawbacks of antibody preparations and select treatments on the basis of a scientific rationale.

As of June 2019, more than 20 patients have been treated with Fasenra^®^ (benralizumab) at our facility, making it an expert facility. We have encountered both benralizumab responders and non-responders in clinical practice.

The primary objective of this study was to explore the predictors of response to benralizumab by reviewing response at week 16 in patients with bronchial asthma and comparing blood test data, subjective symptoms, and respiratory function test results before and after the start of therapy between responders and non-responders. The findings from this study will enable the prediction of response to benralizumab and guide the appropriate use of the drug. This will directly correlate with improvements in the quality of medical care for patients with refractory bronchial asthma, thereby effectively reducing medical costs.

## Methods

The initial study cohort included 39 patients with poorly controlled refractory bronchial asthma who required continuous treatment with oral corticosteroids (OCSs) despite the administration of high-dose inhaled corticosteroids (ICSs), long-acting β-agonists (LABAs), long-acting muscarinic antagonists (LAMAs), leukotriene receptor antagonists (LTRAs), and sustained-released theophylline, as described in asthma treatment step 4 of the Asthma Prevention and Management Guideline 2018.^1^ These patients additionally received 30 mg/dose benralizumab, an anti-interleukin (IL)-5 receptor α monoclonal antibody, as a subcutaneous injection (Fasenra^®^) at weeks 0, 4, and 8 at the Fujita Medical Health University Bantane Hospital between June 2019 and April 2020. In total, 32 patients in the analysis population underwent the asthma control test (ACT), spirography (CHESTAC-8900, Chest M.I., Inc., Tokyo, Japan), measurements of fractional exhaled nitric oxide (NIOX VERO, Chest M.I., Inc.), and blood tests (peripheral eosinophil count, total IgE level) before treatment with benralizumab. Response to treatment was defined (1) an improvement of ≥3 points in the ACT score or (2) a ≥20% reduction in the OCS dose after 16 weeks of benralizumab treatment. On the basis of the collected clinical information, patients were assigned to one of two groups according to the response to benralizumab, and the clinical characteristics of each group were evaluated.

Statistical data analysis was performed using the paired *t*-test, as well as univariate analysis using logistic regression.

The study protocol was approved by the Fujita Health University Medical Research Ethics Committee (HM19-263).

## Results

The baseline characteristics of patients are presented in Table 1.

**Figure 1:**
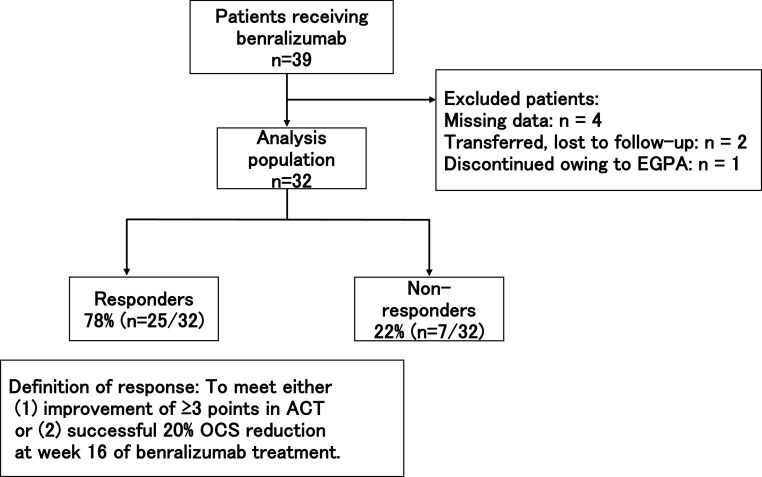
Evaluation at week 16 of benralizumab treatment and baseline characteristics EGPA, eosinophilic granulomatosis with polyangiitis; OCS, oral corticosteroids

In week 16 of benralizumab treatment, 78% (n=25/2) of patients were responders, and 22% (n=7/32) were non-responders (Figure 1).

**Figure 2:**
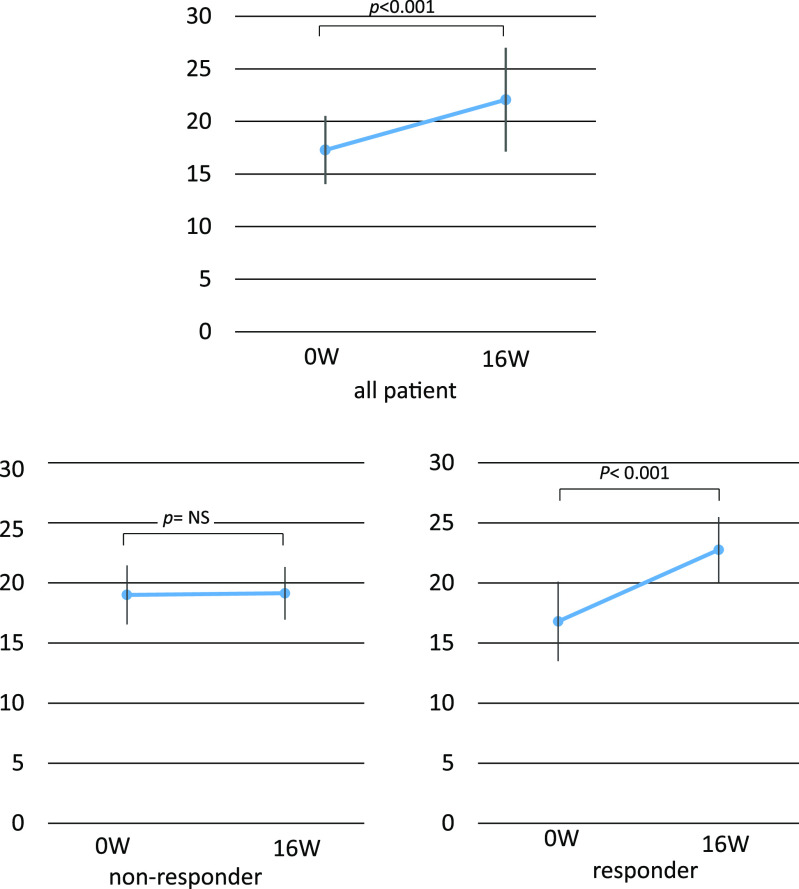
Comparison of ACT between 0 W and 16 W

Relative to baseline, 16 weeks of benralizumab treatment resulted in significant improvements in the ACT score (p<0.001) in responders (Figure 2).

**Figure 3:**
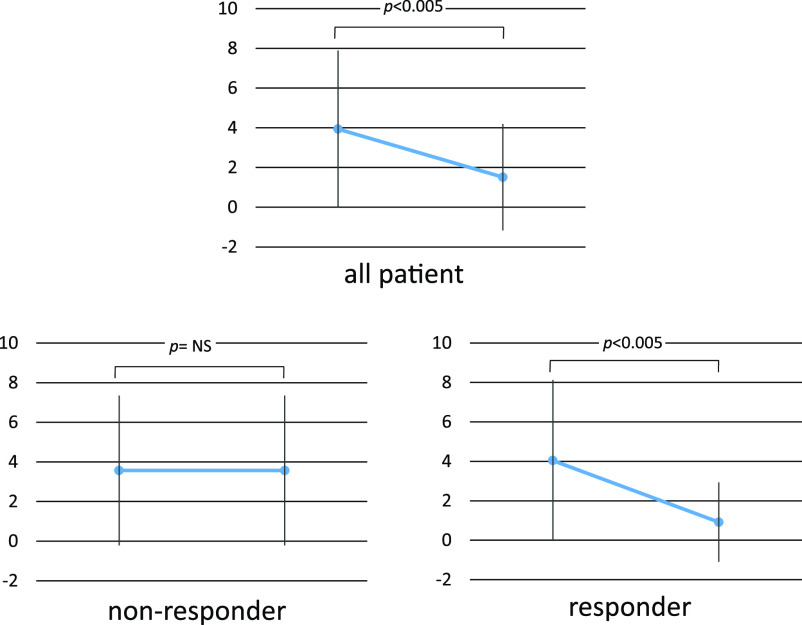
Changes in OCS (mg/d) at 0 W and 16 W

Additionally, 16 weeks of treatment resulted in a ≥20% decline in the OCS dose in responders (p<0.005, Figure 3).

Logistic regression analysis of responders and non-responders after 16 weeks of benralizumab treatment revealed that a peripheral eosinophil count of >300/μL (p<0.001) during benralizumab treatment and a maximal peripheral eosinophil count of >300/μL (p<0.001) in the past year were predictive of response to the drug (Table 2).

## Discussion

Approximately 5%–10% of patients with asthma present with severe refractory asthma requiring the concomitant use of drugs such as high-dose ICSs, LABAs, LAMAs, LTRAs, and theophylline, as well as the continuous use of OCSs.^2,3^ Patients with severe refractory asthma experience impaired quality of life and even death in some cases. In recent years, various antibody preparations have been developed and launched for the treatment of asthma. These antibody preparations have displayed excellent therapeutic benefits when combined with traditional asthma medications. Benralizumab, an anti-IL-5 receptor α monoclonal antibody, was approved for marketing on January 19, 2018 and was launched in April 2018. The underlying mechanism of action of this agent involves the induction of natural killer cells through antibody-dependent cellular cytotoxicity, thereby directly and rapidly removing blood eosinophils, and additionally eliminating sputum and airway eosinophils.

In the Japanese subgroup analysis of the CALIMA study, the annual asthma exacerbation rate was significantly decreased by 83% by treatment compared with the findings in the placebo group.^4^ Although our patients also exhibited a reduced asthma exacerbation rate, this rate was not examined as an indicator of response after 16 weeks of benralizumab treatment in the present study.

In the SIROCCO study, the Asthma Control Questionnaire-6 score was significantly improved after 2 weeks.^5^ In the present study, the changes from baseline to week 16 of benralizumab treatment included a significant improvement in the ACT score (p<0.001).

In the ZONDA study, the daily OCS dose was reduced by 75% from baseline (approximately half of the patients who received dose of ≤12.5 mg withdrew from OCS treatment).^6^ Similar results were observed in the current study, and 78% of patients were considered responders according to the aforementioned criteria.

Furthermore, a subgroup analysis of the SIROCCO and CALIMA studies reportedly predicted response in patients with blood eosinophils ≥150/μL and at least two exacerbations per year, and the results of the ZONDA study predicted response in patients on continuous oral steroid therapy with blood eosinophils counts of ≥150/μL.^6,7^

However, in clinical practice, we have encountered both responders and non-responders to benralizumab among patients with poorly controlled refractory asthma despite adequate treatment with inhaled and oral drugs, a high blood eosinophil count, frequent exacerbations, and continuous OCS use. In this study, patients were evaluated at week 16 of benralizumab treatment. As described previously, although antibody preparations are expensive, predictors of response have not been established, thus making it difficult to examine the advantages and disadvantages of antibody preparations or select antibody preparations based on scientific reasoning. Logistic regression analysis of responders and non-responders at week 16 of benralizumab treatment demonstrated the significance of a peripheral eosinophil count of >300/μL during benralizumab treatment and a maximal eosinophil count of >300/μL in the past year to the response to therapy.

In both the SIROCCO and CALIMA studies, benralizumab significantly improved FEV1 in patients with a peripheral blood eosinophil count of ≥300/μL.^7^

These results suggest that the maximal peripheral eosinophil count in the past year is a promising indicator of response, in addition to the peripheral eosinophil count during benralizumab treatment. Moreover, the possibility that the peripheral blood eosinophil count at baseline may be lower in patients receiving OCS cannot be eliminated. An improved response to benralizumab could be expected using changes in the peripheral blood eosinophil count during the past year as a reference if feasible.

A limited number of studies have investigated predictors of response to benralizumab therapy in patients with refractory bronchial asthma, which was the objective of the present study.

Our results indicated that a peripheral eosinophil count of >300/μL during benralizumab treatment and a maximal peripheral eosinophil count of >300/μL in the past year are predictors of response to the drug. In this study, we investigated the predictors of response only at week 16 of benralizumab treatment; hence, the predictors of response to benralizumab need to be further elucidated by prolonging the study duration and increasing the patient sample size. If the results of this study can guide appropriate benralizumab therapy, the quality of medical care afforded to patients with severe bronchial asthma will be improved, and medical costs will be reduced.

## Figures and Tables

**Table1:** Baseline characteristics of patients

Total n=32	
Age	63.8±17.0
Sex Male:Female	9:23
BMI	24.0±5.1
ACT	17.3±3.2
Steroid bursts in the past 1 year YES:NO	3:29
OCS Yes:No	19:13
Smoking history Yes:No	5:27
Eosinophils	560±303
log IgE	2.3±0.6
FEV_1_	1733±770
FeNO	47.3±35.0

BMI, body mass index; ACT, Asthma Control Test; OCS, oral corticosteroids; IgE, immunoglobulin E; FEV_1_, Forced expiratory volume in 1 second; FeNO, fractional exhaled nitric oxide

**Table2:** Characteristics of responders and non-responders at week 16 of treatment with Fasenra

factors	Odds ratio	95% CI	p-value
Age <65	3.75	0.605–23.3	0.156
Age ≥65	4.44	0.712–27.8	0.111
BMI ≥23	0.131	0.013–1.25	0.078
Smokers	1.14	0.107–12.20	0.912
Steroid bursts in the past 1 year	1.92	0.148–24.9	0.619
At administration of benralizumab Eos >300/μL	15.3	1.91–123.0	0.001
Highest Eos in the past 1 year >300/μL	15.3	1.91–123.0	0.001
log IgE ≥2.3	3.75	0.605–23.30	0.156
FEV_1_(ml) ≥1800	0.40	0.071–2.25	0.298
FeNO(ppb) ≥61	5.71	0.532–61.4	0.150
ATI (%) >1.7%	0.482	0.08–2.68	0.405

Univariate analysis by logistic regression
